# Genome-wide DNA methylation and gene expression analyses in monozygotic twins identify potential biomarkers of depression

**DOI:** 10.1038/s41398-021-01536-y

**Published:** 2021-08-02

**Authors:** Weijing Wang, Weilong Li, Yili Wu, Xiaocao Tian, Haiping Duan, Shuxia Li, Qihua Tan, Dongfeng Zhang

**Affiliations:** 1 Department of Epidemiology and Health Statistics, Public Health College, Qingdao University, Qingdao, Shandong China; 2 Population Research Unit, Faculty of Social Sciences, University of Helsinki, Helsinki, Finland; 3 Qingdao Municipal Center for Disease Control and Prevention/Qingdao Institute of Preventive Medicine, Qingdao, Shandong China; 4 Unit of Human Genetics, Department of Clinical Research, University of Southern Denmark, Odense C, Denmark; 5 Epidemiology and Biostatistics, Department of Public Health, University of Southern Denmark, Odense, Denmark

**Keywords:** Clinical genetics, Personalized medicine

## Abstract

Depression is currently the leading cause of disability around the world. We conducted an epigenome-wide association study (EWAS) in a sample of 58 depression score-discordant monozygotic twin pairs, aiming to detect specific epigenetic variants potentially related to depression and further integrate with gene expression profile data. Association between the methylation level of each CpG site and depression score was tested by applying a linear mixed effect model. Weighted gene co-expression network analysis (WGCNA) was performed for gene expression data. The association of DNA methylation levels of 66 CpG sites with depression score reached the level of *P* < 1 × 10^−4^. These top CpG sites were located at 34 genes, especially *PTPRN2*, *HES5*, *GATA2*, *PRDM7*, and *KCNIP1*. Many ontology enrichments were highlighted, including Notch signaling pathway, Huntington disease, p53 pathway by glucose deprivation, hedgehog signaling pathway, DNA binding, and nucleic acid metabolic process. We detected 19 differentially methylated regions (DMRs), some of which were located at *GRIK2*, *DGKA*, and *NIPA2*. While integrating with gene expression data, *HELZ2*, *PTPRN2*, *GATA2*, and *ZNF624* were differentially expressed. In WGCNA, one specific module was positively correlated with depression score (*r* = 0.62, *P* = 0.002). Some common genes (including *BMP2*, *PRDM7*, *KCNIP1*, and *GRIK2*) and enrichment terms (including complement and coagulation cascades pathway, DNA binding, neuron fate specification, glial cell differentiation, and thyroid gland development) were both identified in methylation analysis and WGCNA. Our study identifies specific epigenetic variations which are significantly involved in regions, functional genes, biological function, and pathways that mediate depression disorder.

## Introduction

Depression is currently the leading cause of disability worldwide [1], and it is predicted to be one of the three leading causes of illness by 2030 [2]. Although the heavy social and economic burden, the potential molecular mechanisms underlying depression remain poorly understood.

The depression risk is influenced by both genetic and environmental factors. It is suggested that epigenetic modification could mediate the lasting increasing depression risk resulting from exposure to adverse life events and provide a mechanistic framework, where genetic and environmental factors were integrated [3, 4]. DNA methylation was one important form of epigenetic modification, and one recent review of 67 studies concluded that there was evidence for the association of DNA methylation variation with depression risk [5]. Additionally, candidate gene studies discovered that *BDNF* and *SLC6A4* hypermethylation were related to depression or major depressive disorder (MDD) [5]. Even currently some significant methylation modifications were found to be associated with depression, however, no consistent results were identified.

Nowadays, using monozygotic twins discordant for a trait or disease has been proved to be a powerful and popular design for EWAS in linking the environmental basis of epigenetic modification variation to disease status while controlling for individual genetic component [6, 7]. This design has been extensively used to explore specific DNA methylation variation associated with diseases, such as cognitive function decline [8], Alzheimer’s disease [9], and rheumatoid arthritis [10]. Since the Chinese population are different from other ethnic populations worldwide concerning genetic constitutions, environmental exposure, a multitude of life styles and occupations, the DNA methylation variation may also differ. However, to our knowledge, yet no EWAS has been performed to explore the DNA methylation variation associated with depression using Chinese monozygotic twin samples.

Accordingly, we aimed to conduct an EWAS to detect DNA CpG sites associated with depression and further integrate with gene expression data in a sample of middle and ole-aged Chinese monozygotic twins.

## Material and methods

The primary materials and methods of this study were similar to our previously published studies [8, 11, 12].

### Participants

Participants recruitment and collection were described in detail previously [13]. Participants who suffered from cerebrovascular disorders, stroke, traumatic brain injury, central nervous system (CNS) tumor, CNS infections, and alcohol or drug dependence were excluded. Meanwhile, participants who were unconscious, unable, or unwilling to cooperate were also dropped. Finally, a total of 58 complete monozygotic twin pairs with a mean age of 52 years (SD: 7.5) were included in the methylation analysis, and a subsample consisted of 12 twin pairs were included in the gene expression analysis. The median of absolute values of intrapair depression score difference (∆(depression score)) of all twins was 4 (range: 1–15). The number of twin pairs for ∆(depression score) ranging in 1–5, 6–10, and 11–15 was 39, 15, and 4 in the methylation analysis and 9, 3, and 0 in the gene expression analysis, respectively. The median of ratio difference calculated as |∆(depression score)|/max(depression score) was 0.41 (range: 0.14–1.00).

After providing written informed consent, all participants took a standardized questionnaire and underwent a health examination. This study was approved by the Regional Ethics Committee of the Qingdao CDC Institutional Review Boards. And the ethical principles of the Helsinki Declaration were also followed.

### Assessment of depression

Depression was assessed by the Geriatric Depression Scale-30 (GDS-30) [14]. The GDS-30 had 30 items, and participants were asked to answer “yes” or “no” to the items based on how they felt over the past 1 week. The higher the total score was, the more severe the participant’s mental condition was.

### Reduced representation bisulfite sequencing (RRBS) analysis

The total DNA was extracted from whole blood and sent to one biomarker technology corporation in China for the RRBS experiment. Briefly, genomic DNA was first digested to generate short fragments that contained CpG dinucleotides at the ends. Then the CpG-rich DNA fragments were extracted and bisulfite-converted. The cDNA library was constructed and sequenced to get raw sequencing data, which was then preprocessed and mapped by *Bismark* [15] and smoothed by R package *BiSeq* [16]. The methylation *β*-value was transformed to *M*-value for statistical modeling. Finally, a total of 551,447 CpG sites were included.

### Cell-type composition estimation

Considering total DNA was extracted from whole blood, different methylation profiles of distinct cell types may lead to false discoveries [17]. We used ReFACTor method to control for the cell-type composition effect on DNA methylation in EWAS [18]. ReFACTor is an unsupervised reference-free method that selects methylation sites, which are informative about the cell composition in the data to apply to principal component analysis (PCA) and further uses the top components of PCA to construct surrogates for the underlying cell-type compositions for adjustment in statistical analysis. In our study, the top five components were chosen as covariates to correct the cell-type heterogeneity.

### RNA library construction, sequencing, and quality control

Briefly, after total mRNA was extracted from whole blood, the RNA-Seq library was constructed and sequenced to get the sequenced data. The data was then mapped to the human genome by TopHat2 [19]. The gene expression level was estimated by FPKM value through Cufflinks software [20].

### Statistical analysis

#### Methylation analysis

##### Epigenome-wide association analysis

The association between the methylation level of each CpG site and depression score was tested by a linear mixed effect model, which was equivalent to the regression model as proposed by Tan et al. [6]. The fixed effect variables of age, gender, and cell-type composition as well as random effect variable of twin pairing were adjusted for in the model.

##### Predicting functions of *cis*-regulatory regions and ontology enrichments analysis

The identified epigenome CpG sites (*P* < 0.05) were submitted to the Genomic Regions Enrichment of Annotations Tool (GREAT) online to analyze the functional significance of *cis*-regulatory regions and ontology enrichments [21]. The default “basal plus extension” association rule was chosen. In this rule, a “basal regulatory region” irrespective of the presence of neighboring genes which extended 5 kb upstream and 1 kb downstream of the transcription start site (TSS) were firstly assigned. Then each gene’s regulatory domain was extended up to the basal regulatory region of the nearest upstream and downstream genes, but no longer than 1 Mb in each direction. FDR < 0.05 was set as statistically significant in ontology enrichments analysis.

##### Differentially methylated region (DMR) analysis

Based on the bisulfite sequencing data and corresponding EWAS results, the DMRs associated with depression score were detected by using the *comb-p* approach [22]. Significant enriched DMRs were evaluated by Stouffer-Liptak-Kechris (*slk*) corrected *P*-value < 0.05.

#### Gene expression analysis

##### Weighted gene co-expression network analysis (WGCNA)

The WGCNA package is a comprehensive collection of R functions for performing various aspects of weighted correlation network analysis [23, 24]. Briefly, we first established a weighted adjacency matrix. Then the topological overlap matrix (TOM) was constructed [25–27] and used as input for hierarchical clustering analysis [28]. Gene modules were detected by using a dynamic tree cutting algorithm. The module eigengenes (MEs) were correlated with the trait of depression score. Relationships among modules were illustrated by a hierarchical clustering dendrogram of MEs [29], and a heatmap plot of the corresponding eigengene network. Intramodular hub genes were defined following criteria of depression score based gene significance (GS) > 0.70 and module membership (MM) > 0.90 with a threshold of *P*-value < 0.01 [30].

For the genes clustered in the module associated with depression score, GO enrichment analysis and BIOCARTA, KEGG, and REACTOME pathway enrichment analysis were conducted by the DAVID tool [31, 32]. The modified fisher exact *P*-value < 0.05 was considered as enrichment cut-off criterion.

##### Differentially expressed genes analysis

Five depression cases (depression score > 10) and eight health controls were included. The gene expression levels of 46 genes (including the genes where the top CpG sites and the DMRs were located) between the two groups were compared by the Wilcoxon rank sum test. The *P*-value < 0.05 was considered as statistically significant.

## Results

### Methylation analysis

A total of 58 monozygotic twin pairs with a mean age of 52 years (SD: 7.5) were included. The median of depression score was 8 (95% range: 0–21). Most of the clinical indicators were statistically intrapair correlated, indicating that the co-twin design could be beneficial (Table 1).

**Table 1:** Basic characteristics of the participants.

Characteristics	Values	Intrapair correlation	
		*r*	*P*-value
Number of twin pairs	58		
*Gender, pairs (%)*			
Male	29 (50)	–	–
Female	29 (50)	–	–
Age, mean (SD) (years)	52 (7.5)	–	–
Depression score, *M* (*P*_2.5_, *P*_97.5_)	8 (0, 21)	0.36	0.006
Cognitive function score, *M* (*P*_2.5_, *P*_97.5_)	21 (8, 30)	0.39*	0.002
BMI, mean (SD), (kg/m^2^)	25.18 (3.63)	0.63**	<0.001
Systolic, *M* (*P*_2.5_, *P*_97.5_) (mmHg)	130 (104, 179)	0.44*	0.001
Diastolic, *M* (*P*_2.5_, *P*_97.5_) (mmHg)	82 (62, 100)	0.31*	0.020
SUA, mean (SD) (μmol/L)	302 (95)	0.52**	<0.001
GLU, M (*P*_2.5_, *P*_97.5_) (mmol/L)	5.4 (3.60, 10.86)	0.57**	<0.001
CHOL, mean (SD) (mmol/L)	4.97 (1.18)	0.54**	<0.001
TG, M (*P*_2.5_, *P*_97.5_) (mmol/L)	1.19 (0.20, 5.67)	0.58**	<0.001
HDLC, M (*P*_2.5_, *P*_97.5_) (mmol/L)	1.34 (0.66, 2.71)	0.83**	<0.001
LDLC, mean (SD) (mmol/L)	2.89 (0.88)	0.45**	<0.001

Continuous variables were presented as mean (standard deviation (SD)) or median (*P*_2.5_, *P*_97.5_); Categorical variables were presented as numbers with percentages.

*BMI* body mass index, *CHOL* total cholesterol, *GLU* fasting glucose, *HDLC* high-density lipoprotein cholesterol, *LDLC* low-density lipoprotein cholesterol, *SUA* serum uric acid, *TG* triglyceride.

### Epigenome-wide association analysis

**Fig. 1:**
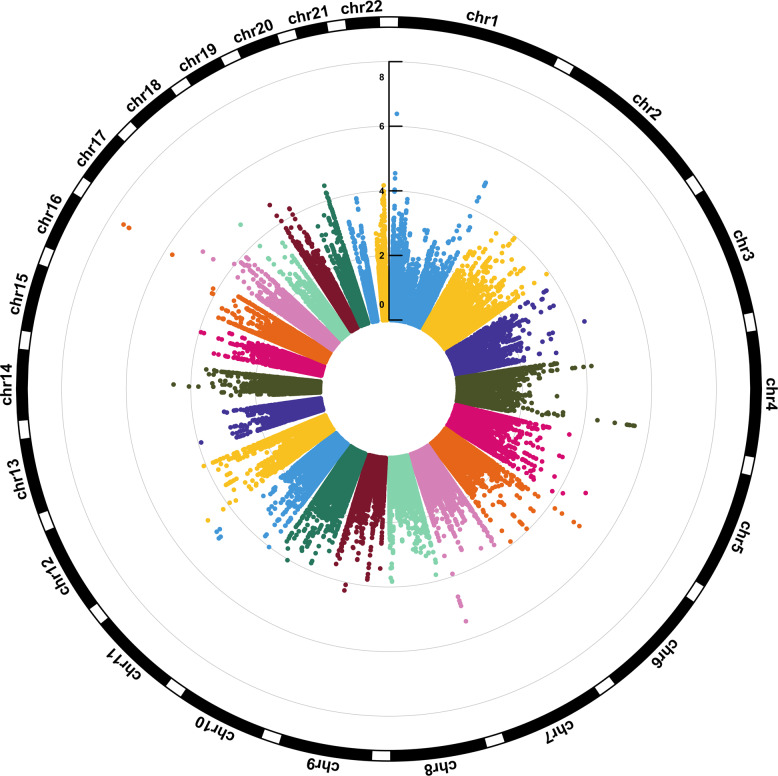
Circular Manhattan plot for epigenome-wide association study of depression. The numbers of chromosome and the −log_10_ of *P*-values for statistical significance are shown. The dots represent the observed CpG sites.

As shown in the Manhattan plot (Fig. 1) and Table 2, the association of DNA methylation levels of 66 top CpG sites with depression score reached the level of *P* < 1 × 10^−4^. The numbers of these top CpG sites located at chr1, chr4, chr16, chr19, chr20, chr11, chr7, chr6, chr12, and chr17 were 9, 8, 7, 6, 6, 5, 5, 4, 3, and 3, respectively. The strongest association (*β* = 0.403, *P* = 2.951 × 10^−8^) was detected for the CpG site (chr16: 90,143,728 bp) located near *PRDM7*. All of the top CpG sites (*P* < 1 × 10^−4^) were located at 34 genes, and there were 5, 4, 4, 4, 3, 3, 3, 3, and 3 CpG sites located at/near *PTPRN2*, *HES5*, *PRDM7*, *RIOK1*, *FCGBP*, *HELZ2*, *HPF1*, *LAMA5*, and *TRIM69*, respectively.

**Table 2:** The results of epigenome-wide association study on depression score (*P*-value <1 × 10^−4^).

Chromosome	Position (bp)	Coefficient	*P*-value	Ensembl gene ID	HGNC symbol
chr16	90,143,728	0.403	2.951E−08	ENSG00000126856	*PRDM7*
chr16	90,143,720	0.402	4.592E−08	ENSG00000126856	*PRDM7*
chr16	90,143,734	0.361	4.714E−08	ENSG00000126856	*PRDM7*
chr1	2,460,431	0.054	4.083E−07	ENSG00000197921	*HES5*
chr16	90,143,752	0.311	1.753E−06	ENSG00000126856	*PRDM7*
chr4	170,696,032	0.132	2.809E−06	NA	NA
chr4	170,696,037	0.131	3.123E−06	NA	NA
chr7	157,794,468	−0.055	3.609E−06	ENSG00000155093	*PTPRN2*
chr4	170,696,018	0.131	4.001E−06	NA	NA
chr4	170,696,053	0.130	4.603E−06	NA	NA
chr4	170,696,014	0.131	4.857E−06	ENSG00000056050	*HPF1*
chr6	7,441,817	0.061	7.444E−06	ENSG00000124784	*RIOK1*
chr17	16,540,145	−0.274	9.447E−06	ENSG00000197566	*ZNF624*
chr7	157,378,324	0.052	1.138E−05	ENSG00000155093	*PTPRN2*
chr4	170,696,062	0.128	1.172E−05	ENSG00000056050	*HPF1*
chr1	231,296,670	0.089	1.202E−05	ENSG00000119283	*TRIM67*
chr6	7,441,838	0.065	1.218E−05	ENSG00000124784	*RIOK1*
chr11	114,480,748	0.041	1.337E−05	ENSG00000137634	*NXPE4*
chr1	231,296,663	0.089	1.358E−05	ENSG00000167333	*TRIM68*
chr7	157,378,313	0.046	1.398E−05	ENSG00000155093	*PTPRN2*
chr1	231,296,660	0.089	1.555E−05	ENSG00000185880	*TRIM69*
chr11	114,480,768	0.044	1.590E−05	ENSG00000137634	*NXPE4*
chr11	130,343,943	0.336	1.617E−05	ENSG00000166106	*ADAMTS15*
chr12	2,903,455	−0.058	1.622E−05	ENSG00000256150	*ITFG2-AS1*
chr7	157,378,307	0.044	1.699E−05	ENSG00000155093	*PTPRN2*
chr5	140,045,268	0.026	1.765E−05	ENSG00000120314	*WDR55*
chr18	18,627,551	−0.040	1.910E−05	ENSG00000124784	*ROCK1*
chr11	130,343,954	0.333	2.152E-05	ENSG00000166106	*ADAMTS15*
chr19	999,961	−0.021	2.233E−05	ENSG00000116032	*GRIN3B*
chr7	157,378,339	0.052	2.274E−05	ENSG00000155093	*PTPRN2*
chr17	16,540,132	−0.270	2.365E−05	ENSG00000197566	*ZNF624*
chr1	2,460,466	0.031	2.831E−05	ENSG00000197921	*HES5*
chr14	59,113,371	0.253	2.918E−05	ENSG00000165617	*DACT1*
chr20	62,194,220	0.206	3.388E−05	ENSG00000130589	*HELZ2*
chr6	7,441,846	0.068	3.433E−05	ENSG00000124784	*RIOK1*
chr1	231,296,648	0.085	3.830E−05	ENSG00000185880	*TRIM69*
chr1	2,475,177	0.062	4.081E−05	ENSG00000197921	*HES5*
chr4	170,696,067	0.127	4.084E−05	ENSG00000056050	*HPF1*
chr1	231,296,641	0.084	5.177E−05	ENSG00000185880	*TRIM69*
chr3	128,205,418	0.142	5.408E−05	ENSG00000179348	*GATA2*
chr19	999,937	−0.019	5.557E−05	ENSG00000116032	*GRIN3B*
chr9^a^	95,376,152	0.272	5.571E−05	ENSG00000188312	*CENPP*
				ENSG00000127080	*IPPK*
chr19	40,366,323	0.163	5.666E−05	ENSG00000275395	*FCGBP*
chr20	60,886,354	0.031	5.690E−05	ENSG00000130702	*LAMA5*
chr20	60,886,357	0.031	5.912E−05	ENSG00000130702	*LAMA5*
chr19	40,366,308	0.163	5.921E−05	ENSG00000275395	*FCGBP*
chr20	62,194,213	0.203	6.059E−05	ENSG00000130589	*HELZ2*
chr12	2,903,464	−0.062	6.221E−05	ENSG00000256150	*ITFG2-AS1*
chr6	7,441,850	0.073	6.404E−05	ENSG00000124784	*RIOK1*
chr22	50,985,408	0.046	6.716E−05	ENSG00000130487	*KLHDC7B*
chr4	24,423,094	0.040	6.749E−05	ENSG00000109819	*PPARGC1A*
chr5	170,068,701	0.044	7.195E−05	ENSG00000182132	*KCNIP1*
chr16	69,969,329	0.249	7.352E−05	ENSG00000198373	*WWP2*
chr20	60,886,348	0.031	7.884E−05	ENSG00000130702	*LAMA5*
chr16	56,998,191	0.040	8.090E−05	ENSG00000087237	*CETP*
chr9^a^	95,376,137	0.283	8.368E−05	ENSG00000188312	*CENPP*
				ENSG00000127080	*IPPK*
chr12	133,341,501	0.039	8.446E−05	ENSG00000176915	*ANKLE2*
chr16	56,998,186	0.038	8.749E−05	ENSG00000087238	*CETP*
chr20	62,194,202	0.202	8.765E−05	ENSG00000130589	*HELZ2*
chr14	54,419,615	0.028	8.799E−05	ENSG00000125378	*BMP4*
chr19	3,823,080	0.041	9.100E−05	ENSG00000105278	*ZFR2*
chr1	2,460,472	0.028	9.116E−05	ENSG00000197921	*HES5*
chr19	40,366,341	0.162	9.234E−05	ENSG00000275395	*FCGBP*
chr10	102,822,675	0.032	9.349E−05	ENSG00000107821	*KAZALD1*
chr17	40,825,688	0.030	9.473E−05	ENSG00000068137	*PLEKHH3*
chr11	2,037,042	0.055	9.810E−05	ENSG00000130600	*H19*

NA not available.

^a^The CpG sites annotated to two genes.

### Predicting functions of *cis*-regulatory regions and ontology enrichments analysis

A total of 15,978 genomic *cis*-regulatory regions were identified to be associated with one or more genes. (Supplementary Fig. 1) Many important pathway terms probably related to depression were significantly enriched, such as Notch signaling pathway, nicotine pharmacodynamics pathway, Huntington disease, p53 pathway by glucose deprivation, Parkinson disease, and hedgehog signaling pathways. Moreover, the GO enriched terms mainly highlighted DNA binding and nucleic acid metabolic process (Table 3).

**Table 3:** The top GREAT ontology enrichments for regions potentially related to depression by using binomial test.

Ontology database	Term name	Binom FDR *Q*-value	Binom region fold enrichment
*Pathways*			
PANTHER	Transcription regulation by bZIP transcription factor	1.94E−24	3.18
PANTHER	Notch signaling pathway	2.38E−07	1.81
PANTHER	Nicotine pharmacodynamics pathway	5.88E−06	1.85
PANTHER	Huntington disease	5.95E−06	1.38
PANTHER	p53 pathway by glucose deprivation	7.56E−05	2.03
PANTHER	ATP synthesis	7.44E−05	10.97
PANTHER	Parkinson disease	7.32E−05	1.45
PANTHER	Hedgehog signaling pathway	1.72E−04	1.76
PANTHER	Muscarinic acetylcholine receptor 1 and 3 signaling pathway	1.31E−03	1.37
PANTHER	Adrenaline and noradrenaline biosynthesis	1.21E−02	1.59
BioCyc	Histidine degradation III	8.26E−09	7.34
BioCyc	Palmitate biosynthesis I (animals)	1.27E−08	3.92
BioCyc	Catecholamine biosynthesis	1.09E−08	6.49
BioCyc	Adenine and adenosine salvage I	2.15E−07	18.12
BioCyc	Aspartate biosynthesis	2.86E−07	8.98
BioCyc	Methylglyoxal degradation VI	4.68E−07	3.41
BioCyc	Oxidized GTP and dGTP detoxification	6.76E−07	18.79
BioCyc	Hypusine biosynthesis	8.58E−07	24.23
BioCyc	Serine and glycine biosynthesis	2.47E−06	3.16
BioCyc	Dolichyl-diphosphooligosaccharide biosynthesis	2.45E−06	3.50
MSigDB	Elongation arrest and recovery	1.03E−43	6.52
MSigDB	Formation of tubulin folding intermediates by CCT/TriC	6.80E−30	5.44
MSigDB	Notch signaling pathway	5.63E−28	2.85
MSigDB	Prefoldin mediated transfer of substrate to CCT/TriC	2.68E−26	4.43
MSigDB	Thrombin signaling through proteinase activated receptors (PARs)	3.13E−25	3.45
MSigDB	Formation of RNA Pol II elongation complex	3.55E−25	3.64
MSigDB	Glucagon signaling in metabolic regulation	1.69E−24	2.84
MSigDB	PKC-catalyzed phosphorylation of inhibitory phosphoprotein of myosin phosphatase	2.20E−23	2.67
MSigDB	Prostacyclin signaling through prostacyclin receptor	4.70E−22	4.40
MSigDB	G alpha (12/13) signaling events	3.12E−20	1.98
*GO function*			
GO-MF	DNA binding	1.26E−114	1.36
GO-MF	Nucleic acid binding	1.06E−97	1.27
GO-MF	Sequence-specific DNA binding transcription factor activity	7.48E−98	1.46
GO-MF	Nucleic acid binding transcription factor activity	9.13E−96	1.46
GO-MF	Sequence-specific DNA binding	1.57E−90	1.54
GO-MF	Organic cyclic compound binding	1.75E−87	1.18
GO-MF	Heterocyclic compound binding	5.68E−87	1.18
GO-MF	Transcription regulatory region DNA binding	4.84E−58	1.61
GO-MF	Regulatory region DNA binding	1.72E−57	1.60
GO-MF	Transcription regulatory region sequence-specific DNA binding	1.64E−53	1.85
GO-BP	RNA metabolic process	1.71E−89	1.27
GO-BP	Gene expression	8.91E−88	1.26
GO-BP	RNA biosynthetic process	7.09E−87	1.30
GO-BP	Transcription, DNA-dependent	7.90E−86	1.30
GO-BP	Regulation of macromolecule biosynthetic process	2.51E−82	1.23
GO-BP	Nucleobase-containing compound biosynthetic process	5.60E−82	1.28
GO-BP	Regulation of RNA biosynthetic process	1.08E−81	1.24
GO-BP	Nucleic acid metabolic process	1.56E−80	1.23
GO-BP	Organic cyclic compound biosynthetic process	2.66E−79	1.26
GO-BP	Regulation of RNA metabolic process	7.78E−79	1.23

*MF* molecular function, *BP* biological process.

### Differentially methylated region (DMR) analysis

**Fig. 2:**
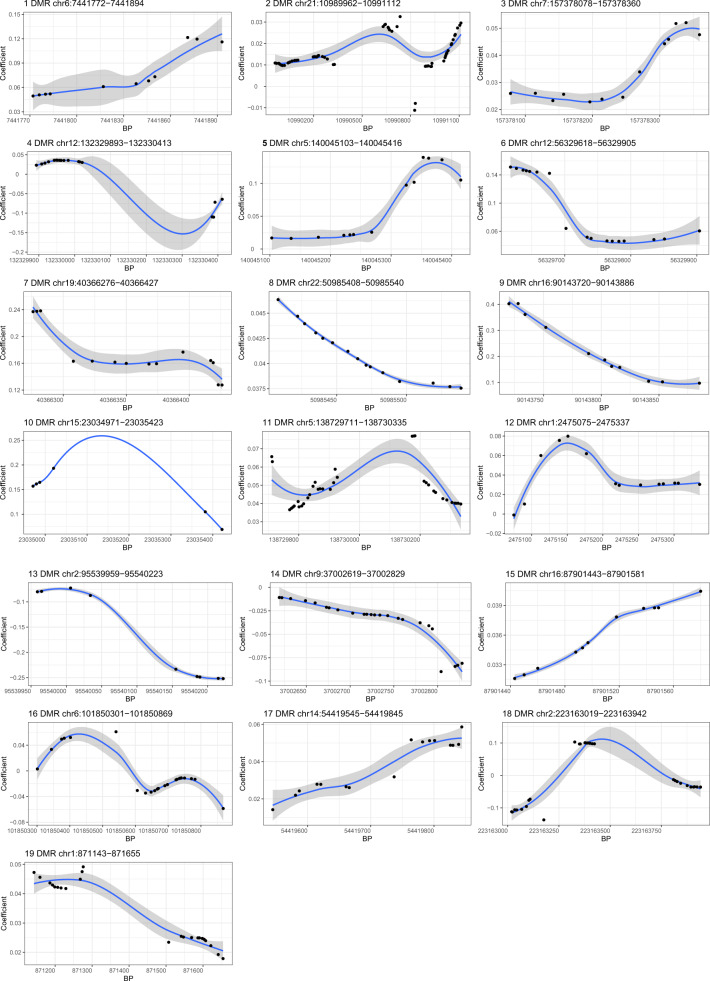
Differential methylation patterns for the identified differentially methylated regions (DMRs). The horizontal axis shows the chromosome positions with the black point indicating each CpG, and the vertical axis shows the coefficient for the association of each CpG sites with depression score. The blue line indicates the methylation pattern for each DMR. BP, base pair; chr, chromosome.

Among the 19 DMRs identified (Fig. 2 and Table 4), the methylation levels of 14 DMRs (1, 2, 3, 5–12, 15, 17, 19) were positively and two DMRs (13, 14) negatively correlated with depression score. But it was difficult to determine the trend of associations between three DMRs (4, 16, 18) and depression score.

**Table 4:** The results of annotation to significant differentially methylated regions (DMRs) (*slk* corrected *P*-value < 0.05).

DMR ID	Chromosome	Start	End	Length	Stouffer-Liptak-Kechris (*slk*) corrected *P*-value	Gene symbol	Location
1	chr6	7,441,772	7,441,894	11	0.001	*CAGE1*	Near
2	chr21	10,989,962	10,991,112	59	0.002	*TPTE*	At
3	chr7	157,378,078	157,378,360	13	0.002	*PTPRN2*	At
4	chr12	132,329,893	132,330,413	18	0.003	*MMP17*	At
5	chr5	140,045,103	140,045,416	13	0.004	*WDR55*	At
6	chr12	56,329,618	56,329,905	17	0.004	*DGKA*	At
7	chr19	40,366,276	40,366,427	14	0.008	*FCGBP*	At
8	chr22	50,985,408	50,985,540	15	0.009	*KLHDC7B*	At
9	chr16	90,143,720	90,143,886	11	0.020	*PRDM7*	At
10	chr15	23,034,971	23,035,423	6	0.021	*NIPA2*	At
11	chr5	138,729,711	138,730,335	36	0.023	*PROB1*	At
12	chr1	2,475,075	2,475,337	14	0.024	*TNFRSF14-AS1*	At
13	chr2	95,539,959	95,540,223	9	0.032	*TEKT4*	At
14	chr9	37,002,619	37,002,829	24	0.036	*PAX5*	At
15	chr16	87,901,443	87,901,581	11	0.040	*SLC7A5*	At
16	chr6	101,850,301	101,850,869	22	0.043	*GRIK2*	At
17	chr14	54,419,545	54,419,845	16	0.044	*BMP4*	At
18	chr2	223,163,019	223,163,942	29	0.045	*PAX3*	At
19	chr1	871,143	871,655	23	0.048	*SAMD11*	At

The DMRs were located at/near 19 genes, among which *DGKA* and *NIPA2* might play an important roles in regulating depression. Interestingly, several DMRs covered the top CpG sites listed in Table 2. The DMR1 (located at *CAGE1*), DMR3 (*PTPRN2*), and DMR9 (*PRDM7*) covered 4 CpG sites, and the DMR7 (located at *FCGBP*) covered three CpG sites.

### Gene expression analysis

There were 12 twin pairs (including seven male pairs) with a median age of 53 years (95% range: 43–65) and a median depression score of 7.5 (range: 1–27) included in the gene expression analysis.

### Weighted gene co-expression network analysis (WGCNA)

**Fig. 3:**
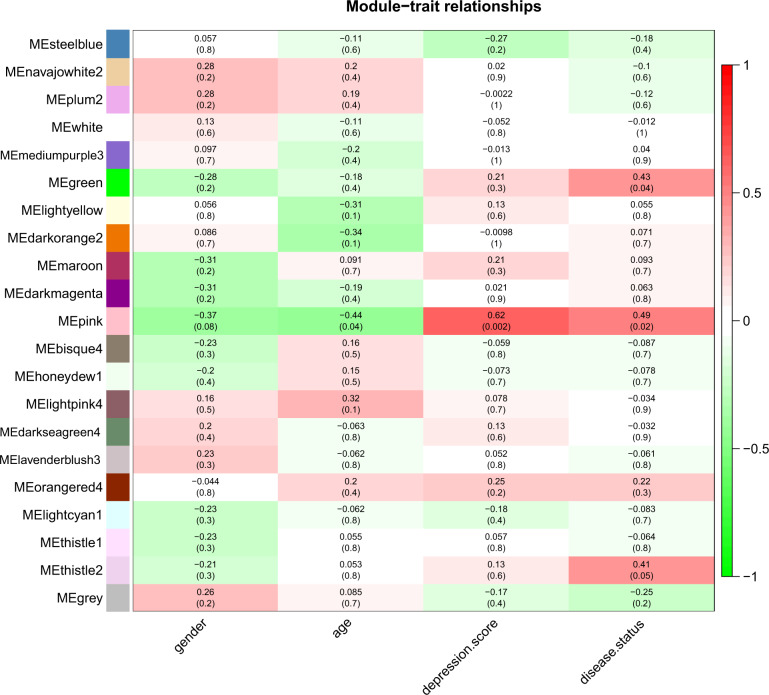
Relationships of consensus module eigengenes and external trait. Each row in the table corresponds to a consensus module, and each column to a trait. Numbers in the table report the correlations of the corresponding module eigengenes and trait with the *P*-values printed below the correlations in parentheses. The table is color coded by correlation according to the color legend.

As Fig. 3 illustrated, genes clustered in pink module (including 3629 genes) were positively correlated with both depression score (*r* = 0.62, *P* = 0.002) and disease status (*r* = 0.49, *P* = 0.02). For this module, neuroactive ligand–receptor interaction, nicotine addiction, calcium signaling pathway, glutam4atergic synapse, and nervous system development were significantly enriched. (Table 5) MM and depression score-based GS exhibited a very significant positive correlation in pink module (*r* = 0.67, *P* < 0.001) (Supplementary Fig. 2), and 27 hub genes were identified (Supplementary Table 1).

**Table 5:** The results of GO function and KEGG pathway enrichment analysis for genes clustered in pink module by DAVID tool.

Category	Term	Count	*P*-value
*Pathways*			
KEGG	Neuroactive ligand–receptor interaction	93	3.03E−14
KEGG	Olfactory transduction	99	5.04E−07
KEGG	Nicotine addiction	20	1.53E−06
KEGG	Calcium signaling pathway	49	4.49E−05
KEGG	Morphine addiction	30	5.49E−05
KEGG	Glutamatergic synapse	35	5.99E−05
KEGG	ECM-receptor interaction	28	1.62E−04
KEGG	Serotonergic synapse	33	1.98E−04
KEGG	Maturity onset diabetes of the young	12	8.83E−04
KEGG	Retrograde endocannabinoid signaling	29	9.61E−04
BIOCARTA	Intrinsic prothrombin activation pathway	10	1.61E−03
KEGG	Bile secretion	21	2.78E−03
KEGG	GABAergic synapse	23	7.88E−03
KEGG	Linoleic acid metabolism	11	8.60E−03
BIOCARTA	Platelet amyloid precursor protein pathway	7	8.88E−03
KEGG	Steroid hormone biosynthesis	17	1.17E−02
KEGG	Complement and coagulation cascades	19	1.42E−02
*GO function*			
GO-BP	Chemical synaptic transmission	88	3.54E−13
GO-BP	Potassium ion transmembrane transport	45	2.07E−07
GO-BP	Regulation of ion transmembrane transport	41	9.55E−07
GO-MF	G-protein coupled receptor activity	167	1.73E−06
GO-MF	Calcium ion binding	165	7.84E−06
GO-MF	Voltage-gated potassium channel activity	24	2.58E−05
GO-BP	G-protein coupled receptor signaling pathway	199	3.06E−05
GO-BP	Sodium ion import across plasma membrane	10	9.99E−05
GO-BP	Nervous system development	74	1.54E−04
GO-BP	G-protein coupled receptor signaling pathway	19	2.83E−04
GO-MF	Glutamate receptor activity	8	6.30E−04
GO-MF	GABA-A receptor activity	10	1.83E−03
GO-BP	Central nervous system development	34	2.46E−03

*MF* molecular function, *BP* biological process.

### The common genes and enrichment terms between methylation analysis and WGCNA

The CpG sites (*P* < 0.05) were annotated to 2808 genes, of which 404 genes were also clustered in the pink module in WGCNA. Among these common genes, *DENND5B*, *KBTBD13*, *TENM3*, and *BMP2* were also identified as hub genes following our strict criteria. And genes including *PRDM7*, *KCNIP1*, *PLEKHH3*, *GRIK2*, *PROB1*, *PAX3* were where the top CpG sites or DMRs were located. (Supplementary Table 2)

Many common enrichment terms were also found, including extra cellular matrix (ECM)-receptor interaction pathway, maturity onset diabetes of the young pathway, complement and coagulation cascades pathway, DNA binding, neuron fate specification, glial cell differentiation, thyroid gland development, and cellular response to hormone stimulus.

### Differentially expressed genes related to depression

Genes including *HELZ2* (*P* = 0.013), *PTPRN2* (*P* = 0.040), *GATA2* (*P* = 0.013), *ZNF624* (*P* = 0.019) were found differentially expressed between the two independent groups.

## Discussion

In this study based on monozygotic twins, we detected some important epigenetic variants underlying depression by EWAS. A total of 66 interesting CpG sites (*P* < 1 × 10^−4^) and 19 DMRs were identified. Moreover, many crucial GREAT ontology enrichments were identified. Genes clustered in the pink module were positively correlated with depression score in WGCNA, and many genes and enrichment terms were overlapped between methylation analysis and WGCNA. Finally, four genes were found to be differentially expressed in depression cases and health controls.

In EWAS, some genes where the top CpG sites and DMRs were located (Tables 2 and 4) may play essential roles in regulating depression status: (1) *PTPRN2*: DNA methylation variation of *PTPRN2* was found to be associated with mood state disturbances across [33]; (2) *HES5*: HES5 could negatively regulate 5-HT1A receptor gene, which was related to MDD and suicide [34]; (3) *GATA2*: It was reported that overexpression of human *GATA2* interfered with spine formation and produced depressive behavior in rats [35]; (4) *DGKA*: Blood transcript levels of *DGKA* differed significantly between participants with MDDs and nondepressed controls [36]; (5) *NIPA2*: It was suggested that rare copy number variants (CNVs) in *NIPA2* could increase the risk of MDD by disrupting regulatory regions [37]; (6) *PRDM7*: The protein encoded by this gene was involved in lysine degradation pathway, and lysine methylation was a physiological post-translational modification of tau protein which played an important role in aging and Alzheimer’s disease [38]; (7) *KCNIP1*: The protein encoded by this gene was a member of the family of cytosolic voltage-gated potassium (Kv) channel-interacting proteins (KCNIPs), and could regulate rapidly inactivating (A-type) currents and hence neuronal membrane excitability; (8) *GRIK2*: *GRIK2*, as one glutamate-related gene, might be related to risk for mood disorders [39], and the gene polymorphism of *GRIK2* was associated with depressive symptoms [40]. The other genes have an unknown function in terms of depression now, whereas they may also be interesting potential candidates to be future researched and validated.

As additional validation, we integrated the methylation data with gene expression data. Genes clustered in the pink module were positively correlated with depression score in WGCNA. And some genes were in common with EWAS findings, like *BMP2*, *PRDM7*, *KCNIP1*, and *GRIK2*. It was indicated that histone deacetylases could control neurogenesis in embryonic brain by inhibiting BMP2/4 signaling [41]. The other three genes had been discussed above. Additionally, four genes *HELZ2*, *PTPRN2*, *GATA2*, and *ZNF624* were differentially expressed between depression cases and health controls. *PTPRN2* and *GATA2* have been discussed above, whereas the biological of *HELZ2* and *ZNF624* involved in depression remained to be studied further.

Two strengths can be noticed in our study. Since the case co-twin design using monozygotic twins discordant for a trait or disease was a powerful tool for EWAS [6, 7], our results based on the twin data would be credible. Meanwhile, considering the various genetic constitutions, environmental exposures, and a multitude of life styles in different ethnic populations worldwide, our findings will specifically help elucidate the underlying pathogenesis of depression in the Chinese population.

Nevertheless, some limitations of our study should also be considered. First, the sample size of the present study was relatively limited due to the challenges of recruiting and identifying qualified twin pairs. We’ll further validate the top CpG sites, essential genes, and biological pathways in a community population. And we’ll also evaluate if the physical distribution of top CpG sites at different chromosomes is over-represented in the regulatory domain of one specific biological pathway. Additionally, we’ll conduct a causal effect analysis based on one specific biological pathway by integrating data of genetic variation, epigenetic variation, and environmental factors. Second, the Townsend deprivation index (TDI) was indicated to be associated with depression [42, 43]. However, we couldn’t add this factor as a covariate in the linear mixed effects model, because we didn’t investigate the corresponding information of TDI during the epidemiological survey. We’ll consider the TDI factor in the validation analysis in the future.

In summary, we confirm that epigenetic factors are significant in explaining depression through twin-based analysis. We detected multiple CpG sites, genes, DMRs, and pathways that were potentially associated with depression. The findings provided an important clues to further elucidate the pathogenesis of depression and helped to identify new diagnostic biomarkers and therapeutic targets for this disease.

## Supplementary information

Supplementary legends

Supplementary table 1

Supplementary table 2

Supplementary figure 1

Supplementary figure 2

## Data Availability

The datasets used and/or analyzed during the current study are available from the corresponding author on reasonable request.
