# Supplementary material for: Genome-wide DNA methylation and gene expression analyses in monozygotic twins identify potential biomarkers of depression
Source: Transl Psychiatry. 2021 Aug 2;11:416. doi: 10.1038/s41398-021-01536-y (PMC8329295; doi:10.1038/s41398-021-01536-y)
Supplement: Supplementary file 1 — Supplementary legends [file 41398_2021_1536_MOESM1_ESM.docx]

**Supplementary materials**

**Supplementary table 1**. The hub genes found by the criterion of depression score-based gene significance (GS) > 0.7 and module membership (MM) > 0.9 with a threshold of *P*-value < 0.01 in pink module.

**Supplementary table 2**. The common genes between methylation analysis and WGCNA.

**Supplementary figure 1**. Number of associated genes per region.

**Supplementary figure 2**. Scatterplots of gene significance (GS) for trait of depression score versus module membership (MM) in the pink module.
