# Supplementary material for: Genome-wide DNA methylation and gene expression analyses in monozygotic twins identify potential biomarkers of depression
Source: Transl Psychiatry. 2021 Aug 2;11:416. doi: 10.1038/s41398-021-01536-y (PMC8329295; doi:10.1038/s41398-021-01536-y)
Supplement: Supplementary file 2 — Supplementary table 1 [file 41398_2021_1536_MOESM2_ESM.docx]

**Supplementary table 1**. The hub genes found by the criterion of depression score-based GS > 0.7 and MM > 0.9 with a threshold of *P*-value < 0.01 in pink module

| **Gene ID** | **Gene symbol** | **MM-pink** | ***P*.MM-pink** | **GS-depression score** | ***P*.GS-depression score** |
| --- | --- | --- | --- | --- | --- |
| ENSG00000112273 | *HDGFL1* | 0.86 | 1.61E-07 | 0.71 | 1.59E-04 |
| ENSG00000120440 | *TTLL2* | 0.87 | 8.36E-08 | 0.72 | 1.24E-04 |
| ENSG00000125845 | *BMP2* | 0.90 | 6.49E-09 | 0.79 | 7.82E-06 |
| ENSG00000140835 | *CHST4* | 0.92 | 3.66E-10 | 0.72 | 1.14E-04 |
| ENSG00000146276 | *GABRR1* | 0.85 | 2.26E-07 | 0.80 | 5.13E-06 |
| ENSG00000146678 | *IGFBP1* | 0.88 | 2.73E-08 | 0.71 | 1.41E-04 |
| ENSG00000152503 | *TRIM36* | 0.85 | 2.84E-07 | 0.73 | 7.22E-05 |
| ENSG00000164035 | *EMCN* | 0.86 | 2.01E-07 | 0.75 | 3.54E-05 |
| ENSG00000165066 | *NKX6-3* | 0.87 | 6.51E-08 | 0.71 | 1.46E-04 |
| ENSG00000170456 | *DENND5B* | 0.80 | 4.52E-06 | 0.72 | 1.07E-04 |
| ENSG00000176198 | *OR11H4* | 0.90 | 6.98E-09 | 0.70 | 1.87E-04 |
| ENSG00000177414 | *UBE2U* | 0.88 | 2.48E-08 | 0.72 | 1.07E-04 |
| ENSG00000179213 | *SIGLECL1* | 0.85 | 2.18E-07 | 0.74 | 5.26E-05 |
| ENSG00000179520 | *SLC17A8* | 0.88 | 2.83E-08 | 0.71 | 1.41E-04 |
| ENSG00000179695 | *OR6C2* | 0.90 | 7.88E-09 | 0.71 | 1.71E-04 |
| ENSG00000180264 | *ADGRD2* | 0.86 | 1.91E-07 | 0.71 | 1.62E-04 |
| ENSG00000183318 | *SPDYE4* | 0.83 | 8.25E-07 | 0.76 | 3.01E-05 |
| ENSG00000188517 | *COL25A1* | 0.88 | 3.28E-08 | 0.71 | 1.29E-04 |
| ENSG00000198390 | *KRTAP13-1* | 0.89 | 1.26E-08 | 0.72 | 1.22E-04 |
| ENSG00000204065 | *TCEAL5* | 0.88 | 3.59E-08 | 0.71 | 1.42E-04 |
| ENSG00000204335 | *SP5* | 0.83 | 9.97E-07 | 0.74 | 5.85E-05 |
| ENSG00000204695 | *OR14J1* | 0.87 | 4.98E-08 | 0.71 | 1.43E-04 |
| ENSG00000214787 | *MS4A4E* | 0.93 | 8.75E-11 | 0.76 | 2.37E-05 |
| ENSG00000218336 | *TENM3* | 0.86 | 1.09E-07 | 0.71 | 1.61E-04 |
| ENSG00000224982 | *TMEM233* | 0.87 | 7.63E-08 | 0.73 | 6.50E-05 |
| ENSG00000234438 | *KBTBD13* | 0.93 | 2.75E-10 | 0.71 | 1.63E-04 |
| ENSG00000241128 | *OR14A2* | 0.88 | 4.17E-08 | 0.71 | 1.42E-04 |

Note: GS: gene significance; MM: module membership
