# Supplementary material for: Genome-wide DNA methylation and gene expression analyses in monozygotic twins identify potential biomarkers of depression
Source: Transl Psychiatry. 2021 Aug 2;11:416. doi: 10.1038/s41398-021-01536-y (PMC8329295; doi:10.1038/s41398-021-01536-y)
Supplement: Supplementary file 3 — Supplementary table 2 [file 41398_2021_1536_MOESM3_ESM.docx]

**Supplementary table 2**. The common genes between methylation analysis and WGCNA

| **Ensembl gene ID** | **HGNC symbol** | **Hub genes in WGCNA** | **Genes where the top CpG was located** | **Genes where the DMRs were located** |
| --- | --- | --- | --- | --- |
| ENSG00000126856 | *PRDM7* |  | Yes | Yes |
| ENSG00000182132 | *KCNIP1* |  | Yes |  |
| ENSG00000068137 | *PLEKHH3* |  | Yes |  |
| ENSG00000164418 | *GRIK2* |  |  | Yes |
| ENSG00000182255 | *KCNA4* |  |  |  |
| ENSG00000176723 | *ZNF843* |  |  |  |
| ENSG00000170456 | *DENND5B* | Yes |  |  |
| ENSG00000111046 | *MYF6* |  |  |  |
| ENSG00000078900 | *TP73* |  |  |  |
| ENSG00000148408 | *CACNA1B* |  |  |  |
| ENSG00000177096 | *FAM109B* |  |  |  |
| ENSG00000174672 | *BRSK2* |  |  |  |
| ENSG00000174963 | *ZIC4* |  |  |  |
| ENSG00000172901 | NA |  |  |  |
| ENSG00000116151 | *MORN1* |  |  |  |
| ENSG00000168348 | *INSM2* |  |  |  |
| ENSG00000125851 | *PCSK2* |  |  |  |
| ENSG00000182107 | *TMEM30B* |  |  |  |
| ENSG00000206579 | *XKR4* |  |  |  |
| ENSG00000178342 | *KCNG2* |  |  |  |
| ENSG00000111199 | *TRPV4* |  |  |  |
| ENSG00000102901 | *CENPT* |  |  |  |
| ENSG00000184486 | *POU3F2* |  |  |  |
| ENSG00000118946 | *PCDH17* |  |  |  |
| ENSG00000179776 | *CDH5* |  |  |  |
| ENSG00000167291 | *TBC1D16* |  |  |  |
| ENSG00000113212 | *PCDHB7* |  |  |  |
| ENSG00000165443 | *PHYHIPL* |  |  |  |
| ENSG00000129910 | *CDH15* |  |  |  |
| ENSG00000134278 | *SPIRE1* |  |  |  |
| ENSG00000168505 | *GBX2* |  |  |  |
| ENSG00000116833 | *NR5A2* |  |  |  |
| ENSG00000185823 | *NPAP1* |  |  |  |
| ENSG00000181072 | *CHRM2* |  |  |  |
| ENSG00000104901 | *DKKL1* |  |  |  |
| ENSG00000196967 | *ZNF585A* |  |  |  |
| ENSG00000106689 | *LHX2* |  |  |  |
| ENSG00000125798 | *FOXA2* |  |  |  |
| ENSG00000228672 | *PROB1* |  |  | Yes |
| ENSG00000099999 | *RNF215* |  |  |  |
| ENSG00000099625 | *C19orf26* |  |  |  |
| ENSG00000183654 | NA |  |  |  |
| ENSG00000160539 | *PPAPDC3* |  |  |  |
| ENSG00000150551 | *LYPD1* |  |  |  |
| ENSG00000172572 | *PDE3A* |  |  |  |
| ENSG00000169676 | *DRD5* |  |  |  |
| ENSG00000080709 | *KCNN2* |  |  |  |
| ENSG00000129514 | *FOXA1* |  |  |  |
| ENSG00000101746 | *NOL4* |  |  |  |
| ENSG00000141556 | *TBCD* |  |  |  |
| ENSG00000150051 | *MKX* |  |  |  |
| ENSG00000141101 | *NOB1* |  |  |  |
| ENSG00000121653 | *MAPK8IP1* |  |  |  |
| ENSG00000163288 | *GABRB1* |  |  |  |
| ENSG00000196132 | *MYT1* |  |  |  |
| ENSG00000137857 | *DUOX1* |  |  |  |
| ENSG00000187486 | *KCNJ11* |  |  |  |
| ENSG00000007372 | *PAX6* |  |  |  |
| ENSG00000173838 | NA |  |  |  |
| ENSG00000128652 | *HOXD3* |  |  |  |
| ENSG00000161896 | *IP6K3* |  |  |  |
| ENSG00000213578 | *CPLX3* |  |  |  |
| ENSG00000122966 | *CIT* |  |  |  |
| ENSG00000213023 | *SYT3* |  |  |  |
| ENSG00000225556 | *C2CD4D* |  |  |  |
| ENSG00000151789 | *ZNF385D* |  |  |  |
| ENSG00000197162 | *ZNF785* |  |  |  |
| ENSG00000173421 | *CCDC36* |  |  |  |
| ENSG00000107147 | *KCNT1* |  |  |  |
| ENSG00000234438 | *KBTBD13* | Yes |  |  |
| ENSG00000162714 | *ZNF496* |  |  |  |
| ENSG00000060709 | *RIMBP2* |  |  |  |
| ENSG00000159212 | *CLIC6* |  |  |  |
| ENSG00000108001 | *EBF3* |  |  |  |
| ENSG00000131864 | *USP29* |  |  |  |
| ENSG00000147799 | *ARHGAP39* |  |  |  |
| ENSG00000182771 | *GRID1* |  |  |  |
| ENSG00000171729 | *TMEM51* |  |  |  |
| ENSG00000175497 | *DPP10* |  |  |  |
| ENSG00000099822 | *HCN2* |  |  |  |
| ENSG00000222017 | NA |  |  |  |
| ENSG00000112246 | *SIM1* |  |  |  |
| ENSG00000169184 | *MN1* |  |  |  |
| ENSG00000170370 | *EMX2* |  |  |  |
| ENSG00000130675 | *MNX1* |  |  |  |
| ENSG00000155897 | *ADCY8* |  |  |  |
| ENSG00000144063 | *MALL* |  |  |  |
| ENSG00000140279 | *DUOX2* |  |  |  |
| ENSG00000178403 | *NEUROG2* |  |  |  |
| ENSG00000181234 | *TMEM132C* |  |  |  |
| ENSG00000106404 | *CLDN15* |  |  |  |
| ENSG00000139537 | *CCDC65* |  |  |  |
| ENSG00000151136 | *BTBD11* |  |  |  |
| ENSG00000186188 | *FFAR4* |  |  |  |
| ENSG00000138207 | *RBP4* |  |  |  |
| ENSG00000137124 | *ALDH1B1* |  |  |  |
| ENSG00000113657 | *DPYSL3* |  |  |  |
| ENSG00000112877 | *CEP72* |  |  |  |
| ENSG00000126217 | *MCF2L* |  |  |  |
| ENSG00000187140 | *FOXD3* |  |  |  |
| ENSG00000196876 | *SCN8A* |  |  |  |
| ENSG00000134917 | *ADAMTS8* |  |  |  |
| ENSG00000120093 | *HOXB3* |  |  |  |
| ENSG00000143768 | *LEFTY2* |  |  |  |
| ENSG00000197653 | *DNAH10* |  |  |  |
| ENSG00000122778 | *KIAA1549* |  |  |  |
| ENSG00000068078 | *FGFR3* |  |  |  |
| ENSG00000170893 | *TRH* |  |  |  |
| ENSG00000109610 | *SOD3* |  |  |  |
| ENSG00000124140 | *SLC12A5* |  |  |  |
| ENSG00000130226 | *DPP6* |  |  |  |
| ENSG00000214357 | *NEURL1B* |  |  |  |
| ENSG00000180318 | *ALX1* |  |  |  |
| ENSG00000168993 | *CPLX1* |  |  |  |
| ENSG00000162105 | *SHANK2* |  |  |  |
| ENSG00000179148 | *ALOXE3* |  |  |  |
| ENSG00000063660 | *GPC1* |  |  |  |
| ENSG00000172554 | *SNTG2* |  |  |  |
| ENSG00000128805 | *ARHGAP22* |  |  |  |
| ENSG00000174950 | *CD164L2* |  |  |  |
| ENSG00000176381 | *PRR18* |  |  |  |
| ENSG00000187240 | *DYNC2H1* |  |  |  |
| ENSG00000101198 | *NKAIN4* |  |  |  |
| ENSG00000121742 | *GJB6* |  |  |  |
| ENSG00000204956 | *PCDHGA1* |  |  |  |
| ENSG00000081853 | *PCDHGA2* |  |  |  |
| ENSG00000196277 | *GRM7* |  |  |  |
| ENSG00000107902 | *LHPP* |  |  |  |
| ENSG00000163808 | *KIF15* |  |  |  |
| ENSG00000163071 | *SPATA18* |  |  |  |
| ENSG00000178187 | *ZNF454* |  |  |  |
| ENSG00000095203 | *EPB41L4B* |  |  |  |
| ENSG00000125531 | *C20orf195* |  |  |  |
| ENSG00000153495 | *TEX29* |  |  |  |
| ENSG00000141391 | *SLMO1* |  |  |  |
| ENSG00000154277 | *UCHL1* |  |  |  |
| ENSG00000128159 | *TUBGCP6* |  |  |  |
| ENSG00000074047 | *GLI2* |  |  |  |
| ENSG00000042813 | *ZPBP* |  |  |  |
| ENSG00000163618 | *CADPS* |  |  |  |
| ENSG00000091010 | *POU4F3* |  |  |  |
| ENSG00000105784 | *RUNDC3B* |  |  |  |
| ENSG00000101187 | *SLCO4A1* |  |  |  |
| ENSG00000099617 | *EFNA2* |  |  |  |
| ENSG00000142615 | *CELA2A* |  |  |  |
| ENSG00000071242 | *RPS6KA2* |  |  |  |
| ENSG00000198796 | *ALPK2* |  |  |  |
| ENSG00000161082 | *CELF5* |  |  |  |
| ENSG00000146090 | *RASGEF1C* |  |  |  |
| ENSG00000164379 | *FOXQ1* |  |  |  |
| ENSG00000154118 | *JPH3* |  |  |  |
| ENSG00000083457 | *ITGAE* |  |  |  |
| ENSG00000104825 | *NFKBIB* |  |  |  |
| ENSG00000248405 | *PRR5-ARHGAP8* |  |  |  |
| ENSG00000006638 | *TBXA2R* |  |  |  |
| ENSG00000185551 | *NR2F2* |  |  |  |
| ENSG00000169851 | *PCDH7* |  |  |  |
| ENSG00000188730 | *VWC2* |  |  |  |
| ENSG00000100027 | *YPEL1* |  |  |  |
| ENSG00000113205 | *PCDHB3* |  |  |  |
| ENSG00000163347 | *CLDN1* |  |  |  |
| ENSG00000114473 | *IQCG* |  |  |  |
| ENSG00000155052 | *CNTNAP5* |  |  |  |
| ENSG00000151338 | *MIPOL1* |  |  |  |
| ENSG00000156885 | *COX6A2* |  |  |  |
| ENSG00000171885 | *AQP4* |  |  |  |
| ENSG00000102924 | *CBLN1* |  |  |  |
| ENSG00000215217 | *C5orf49* |  |  |  |
| ENSG00000107623 | *GDF10* |  |  |  |
| ENSG00000075391 | *RASAL2* |  |  |  |
| ENSG00000105880 | *DLX5* |  |  |  |
| ENSG00000160886 | *LY6K* |  |  |  |
| ENSG00000176697 | *BDNF* |  |  |  |
| ENSG00000066230 | *SLC9A3* |  |  |  |
| ENSG00000166206 | *GABRB3* |  |  |  |
| ENSG00000182156 | *ENPP7* |  |  |  |
| ENSG00000111490 | *TBC1D30* |  |  |  |
| ENSG00000166183 | *ASPG* |  |  |  |
| ENSG00000175911 | NA |  |  |  |
| ENSG00000117400 | *MPL* |  |  |  |
| ENSG00000135903 | *PAX3* |  |  | Yes |
| ENSG00000137203 | *TFAP2A* |  |  |  |
| ENSG00000148297 | *MED22* |  |  |  |
| ENSG00000251322 | *SHANK3* |  |  |  |
| ENSG00000168569 | *TMEM223* |  |  |  |
| ENSG00000163702 | *IL17RC* |  |  |  |
| ENSG00000179403 | *VWA1* |  |  |  |
| ENSG00000198300 | *PEG3* |  |  |  |
| ENSG00000204970 | *PCDHA1* |  |  |  |
| ENSG00000204969 | *PCDHA2* |  |  |  |
| ENSG00000204965 | *PCDHA5* |  |  |  |
| ENSG00000081842 | *PCDHA6* |  |  |  |
| ENSG00000204963 | *PCDHA7* |  |  |  |
| ENSG00000172789 | *HOXC5* |  |  |  |
| ENSG00000165970 | *SLC6A5* |  |  |  |
| ENSG00000182747 | *SLC35D3* |  |  |  |
| ENSG00000140092 | *FBLN5* |  |  |  |
| ENSG00000130758 | *MAP3K10* |  |  |  |
| ENSG00000139915 | *MDGA2* |  |  |  |
| ENSG00000129347 | *KRI1* |  |  |  |
| ENSG00000218336 | *TENM3* | Yes |  |  |
| ENSG00000186197 | *EDARADD* |  |  |  |
| ENSG00000122417 | *ODF2L* |  |  |  |
| ENSG00000170160 | *CCDC144A* |  |  |  |
| ENSG00000185760 | *KCNQ5* |  |  |  |
| ENSG00000101331 | *CCM2L* |  |  |  |
| ENSG00000163633 | *C4orf36* |  |  |  |
| ENSG00000187372 | *PCDHB13* |  |  |  |
| ENSG00000178882 | *FAM101A* |  |  |  |
| ENSG00000141448 | *GATA6* |  |  |  |
| ENSG00000218672 | NA |  |  |  |
| ENSG00000196711 | *FAM150A* |  |  |  |
| ENSG00000105997 | *HOXA3* |  |  |  |
| ENSG00000179528 | *LBX2* |  |  |  |
| ENSG00000147647 | *DPYS* |  |  |  |
| ENSG00000061455 | *PRDM6* |  |  |  |
| ENSG00000158220 | *ESYT3* |  |  |  |
| ENSG00000103269 | *RHBDL1* |  |  |  |
| ENSG00000163032 | *VSNL1* |  |  |  |
| ENSG00000142178 | *SIK1* |  |  |  |
| ENSG00000198010 | *DLGAP2* |  |  |  |
| ENSG00000165556 | *CDX2* |  |  |  |
| ENSG00000120329 | *SLC25A2* |  |  |  |
| ENSG00000102539 | *MLNR* |  |  |  |
| ENSG00000134627 | *PIWIL4* |  |  |  |
| ENSG00000170616 | *SCRT1* |  |  |  |
| ENSG00000179915 | *NRXN1* |  |  |  |
| ENSG00000206069 | *TMEM211* |  |  |  |
| ENSG00000099381 | *SETD1A* |  |  |  |
| ENSG00000148602 | *LRIT1* |  |  |  |
| ENSG00000104967 | *NOVA2* |  |  |  |
| ENSG00000179603 | *GRM8* |  |  |  |
| ENSG00000160224 | *AIRE* |  |  |  |
| ENSG00000142511 | *GPR32* |  |  |  |
| ENSG00000009950 | *MLXIPL* |  |  |  |
| ENSG00000156966 | *B3GNT7* |  |  |  |
| ENSG00000165553 | *NGB* |  |  |  |
| ENSG00000100095 | *SEZ6L* |  |  |  |
| ENSG00000198597 | *ZNF536* |  |  |  |
| ENSG00000161267 | *BDH1* |  |  |  |
| ENSG00000169047 | *IRS1* |  |  |  |
| ENSG00000173614 | *NMNAT1* |  |  |  |
| ENSG00000125337 | *KIF25* |  |  |  |
| ENSG00000184937 | *WT1* |  |  |  |
| ENSG00000164236 | *ANKRD33B* |  |  |  |
| ENSG00000186047 | *DLEU7* |  |  |  |
| ENSG00000136574 | *GATA4* |  |  |  |
| ENSG00000169783 | *LINGO1* |  |  |  |
| ENSG00000171227 | *TMEM37* |  |  |  |
| ENSG00000198369 | *SPRED2* |  |  |  |
| ENSG00000081923 | *ATP8B1* |  |  |  |
| ENSG00000077063 | *CTTNBP2* |  |  |  |
| ENSG00000087460 | *GNAS* |  |  |  |
| ENSG00000104499 | *GML* |  |  |  |
| ENSG00000160882 | *CYP11B1* |  |  |  |
| ENSG00000135925 | *WNT10A* |  |  |  |
| ENSG00000130201 | *EXOC3L2* |  |  |  |
| ENSG00000171126 | *KCNG3* |  |  |  |
| ENSG00000177103 | *DSCAML1* |  |  |  |
| ENSG00000072163 | *LIMS2* |  |  |  |
| ENSG00000125731 | *SH2D3A* |  |  |  |
| ENSG00000138336 | *TET1* |  |  |  |
| ENSG00000170579 | *DLGAP1* |  |  |  |
| ENSG00000154856 | *APCDD1* |  |  |  |
| ENSG00000118160 | *SLC8A2* |  |  |  |
| ENSG00000164093 | *PITX2* |  |  |  |
| ENSG00000206075 | *SERPINB5* |  |  |  |
| ENSG00000188523 | *C9orf171* |  |  |  |
| ENSG00000156011 | *PSD3* |  |  |  |
| ENSG00000213213 | *CCDC183* |  |  |  |
| ENSG00000169840 | *GSX1* |  |  |  |
| ENSG00000148840 | *PPRC1* |  |  |  |
| ENSG00000183072 | *NKX2-5* |  |  |  |
| ENSG00000139209 | *SLC38A4* |  |  |  |
| ENSG00000215912 | *TTC34* |  |  |  |
| ENSG00000067715 | *SYT1* |  |  |  |
| ENSG00000139515 | *PDX1* |  |  |  |
| ENSG00000165323 | *FAT3* |  |  |  |
| ENSG00000163069 | *SGCB* |  |  |  |
| ENSG00000188227 | *ZNF793* |  |  |  |
| ENSG00000008735 | *MAPK8IP2* |  |  |  |
| ENSG00000146555 | *SDK1* |  |  |  |
| ENSG00000124120 | *TTPAL* |  |  |  |
| ENSG00000089225 | *TBX5* |  |  |  |
| ENSG00000165084 | *C8orf34* |  |  |  |
| ENSG00000224940 | *PRRT4* |  |  |  |
| ENSG00000154736 | *ADAMTS5* |  |  |  |
| ENSG00000091622 | *PITPNM3* |  |  |  |
| ENSG00000178919 | *FOXE1* |  |  |  |
| ENSG00000109686 | *SH3D19* |  |  |  |
| ENSG00000180447 | *GAS1* |  |  |  |
| ENSG00000078295 | *ADCY2* |  |  |  |
| ENSG00000065618 | *COL17A1* |  |  |  |
| ENSG00000114790 | *ARHGEF26* |  |  |  |
| ENSG00000188677 | *PARVB* |  |  |  |
| ENSG00000136099 | *PCDH8* |  |  |  |
| ENSG00000145147 | *SLIT2* |  |  |  |
| ENSG00000149256 | *TENM4* |  |  |  |
| ENSG00000006453 | *BAIAP2L1* |  |  |  |
| ENSG00000131037 | *EPS8L1* |  |  |  |
| ENSG00000118257 | *NRP2* |  |  |  |
| ENSG00000197651 | *CCER1* |  |  |  |
| ENSG00000204128 | *C2orf72* |  |  |  |
| ENSG00000148156 | *ACTL7B* |  |  |  |
| ENSG00000112232 | *KHDRBS2* |  |  |  |
| ENSG00000171385 | *KCND3* |  |  |  |
| ENSG00000196166 | *C8orf86* |  |  |  |
| ENSG00000125398 | *SOX9* |  |  |  |
| ENSG00000186790 | *FOXE3* |  |  |  |
| ENSG00000050438 | *SLC4A8* |  |  |  |
| ENSG00000170004 | *CHD3* |  |  |  |
| ENSG00000036828 | *CASR* |  |  |  |
| ENSG00000113763 | *UNC5A* |  |  |  |
| ENSG00000186976 | *EFCAB6* |  |  |  |
| ENSG00000250120 | *PCDHA10* |  |  |  |
| ENSG00000251664 | *PCDHA12* |  |  |  |
| ENSG00000053438 | *NNAT* |  |  |  |
| ENSG00000145975 | *FAM217A* |  |  |  |
| ENSG00000181786 | *ACTL9* |  |  |  |
| ENSG00000040531 | *CTNS* |  |  |  |
| ENSG00000152822 | *GRM1* |  |  |  |
| ENSG00000109576 | *AADAT* |  |  |  |
| ENSG00000123388 | *HOXC11* |  |  |  |
| ENSG00000161509 | *GRIN2C* |  |  |  |
| ENSG00000145945 | *FAM50B* |  |  |  |
| ENSG00000165059 | *PRKACG* |  |  |  |
| ENSG00000184058 | *TBX1* |  |  |  |
| ENSG00000233608 | *TWIST2* |  |  |  |
| ENSG00000141255 | *SPATA22* |  |  |  |
| ENSG00000176887 | *SOX11* |  |  |  |
| ENSG00000144451 | *SPAG16* |  |  |  |
| ENSG00000174990 | *CA5A* |  |  |  |
| ENSG00000130707 | *ASS1* |  |  |  |
| ENSG00000163251 | *FZD5* |  |  |  |
| ENSG00000176749 | *CDK5R1* |  |  |  |
| ENSG00000138039 | *LHCGR* |  |  |  |
| ENSG00000155918 | *RAET1L* |  |  |  |
| ENSG00000088808 | *PPP1R13B* |  |  |  |
| ENSG00000134533 | *RERG* |  |  |  |
| ENSG00000134640 | *MTNR1B* |  |  |  |
| ENSG00000145506 | *NKD2* |  |  |  |
| ENSG00000119888 | *EPCAM* |  |  |  |
| ENSG00000140009 | *ESR2* |  |  |  |
| ENSG00000105137 | *SYDE1* |  |  |  |
| ENSG00000157077 | *ZFYVE9* |  |  |  |
| ENSG00000005073 | *HOXA11* |  |  |  |
| ENSG00000111218 | *PRMT8* |  |  |  |
| ENSG00000176165 | *FOXG1* |  |  |  |
| ENSG00000037280 | *FLT4* |  |  |  |
| ENSG00000235608 | *NKX1-1* |  |  |  |
| ENSG00000107562 | *CXCL12* |  |  |  |
| ENSG00000136718 | *IMP4* |  |  |  |
| ENSG00000184702 | NA |  |  |  |
| ENSG00000078549 | *ADCYAP1R1* |  |  |  |
| ENSG00000178828 | *RNF186* |  |  |  |
| ENSG00000119771 | *KLHL29* |  |  |  |
| ENSG00000189325 | *C6orf222* |  |  |  |
| ENSG00000226174 | *TEX22* |  |  |  |
| ENSG00000173480 | *ZNF417* |  |  |  |
| ENSG00000162944 | *RFTN2* |  |  |  |
| ENSG00000170549 | *IRX1* |  |  |  |
| ENSG00000171219 | *CDC42BPG* |  |  |  |
| ENSG00000183473 | *SSTR3* |  |  |  |
| ENSG00000219438 | *FAM19A5* |  |  |  |
| ENSG00000143951 | *WDPCP* |  |  |  |
| ENSG00000196090 | *PTPRT* |  |  |  |
| ENSG00000103546 | *SLC6A2* |  |  |  |
| ENSG00000173210 | *ABLIM3* |  |  |  |
| ENSG00000196367 | *TRRAP* |  |  |  |
| ENSG00000104369 | *JPH1* |  |  |  |
| ENSG00000167107 | *ACSF2* |  |  |  |
| ENSG00000173531 | *MST1* |  |  |  |
| ENSG00000175928 | *LRRN1* |  |  |  |
| ENSG00000105971 | *CAV2* |  |  |  |
| ENSG00000129946 | *SHC2* |  |  |  |
| ENSG00000137801 | *THBS1* |  |  |  |
| ENSG00000066382 | *MPPED2* |  |  |  |
| ENSG00000125845 | *BMP2* | Yes |  |  |
| ENSG00000043355 | *ZIC2* |  |  |  |
| ENSG00000112541 | *PDE10A* |  |  |  |
| ENSG00000196208 | *GREB1* |  |  |  |
| ENSG00000163499 | *CRYBA2* |  |  |  |
| ENSG00000154485 | *MMP21* |  |  |  |
| ENSG00000198719 | *DLL1* |  |  |  |
| ENSG00000168487 | *BMP1* |  |  |  |
| ENSG00000173826 | *KCNH6* |  |  |  |
| ENSG00000130338 | *TULP4* |  |  |  |
| ENSG00000185361 | *TNFAIP8L1* |  |  |  |
| ENSG00000167476 | *JSRP1* |  |  |  |
| ENSG00000100154 | *TTC28* |  |  |  |
| ENSG00000213889 | *PPM1N* |  |  |  |
| ENSG00000153303 | *FRMD1* |  |  |  |
| ENSG00000124159 | *MATN4* |  |  |  |
| ENSG00000108018 | *SORCS1* |  |  |  |
| ENSG00000182379 | *NXPH4* |  |  |  |
| ENSG00000114654 | *EFCC1* |  |  |  |
| ENSG00000136378 | *ADAMTS7* |  |  |  |
| ENSG00000074410 | *CA12* |  |  |  |
| ENSG00000162510 | *MATN1* |  |  |  |
| ENSG00000197826 | *C4orf22* |  |  |  |
| ENSG00000183230 | *CTNNA3* |  |  |  |
| ENSG00000109851 | *DBX1* |  |  |  |
| ENSG00000187848 | *P2RX2* |  |  |  |
| ENSG00000184154 | *LRTOMT* |  |  |  |

Note: DMR, differentially methylated region; NA, not available
