# Supplementary material for: Genome-wide DNA methylation and gene expression analyses in monozygotic twins identify potential biomarkers of depression
Source: Transl Psychiatry. 2021 Aug 2;11:416. doi: 10.1038/s41398-021-01536-y (PMC8329295; doi:10.1038/s41398-021-01536-y)
Supplement: Supplementary file 4 — Supplementary figure 1 [file 41398_2021_1536_MOESM4_ESM.pdf]

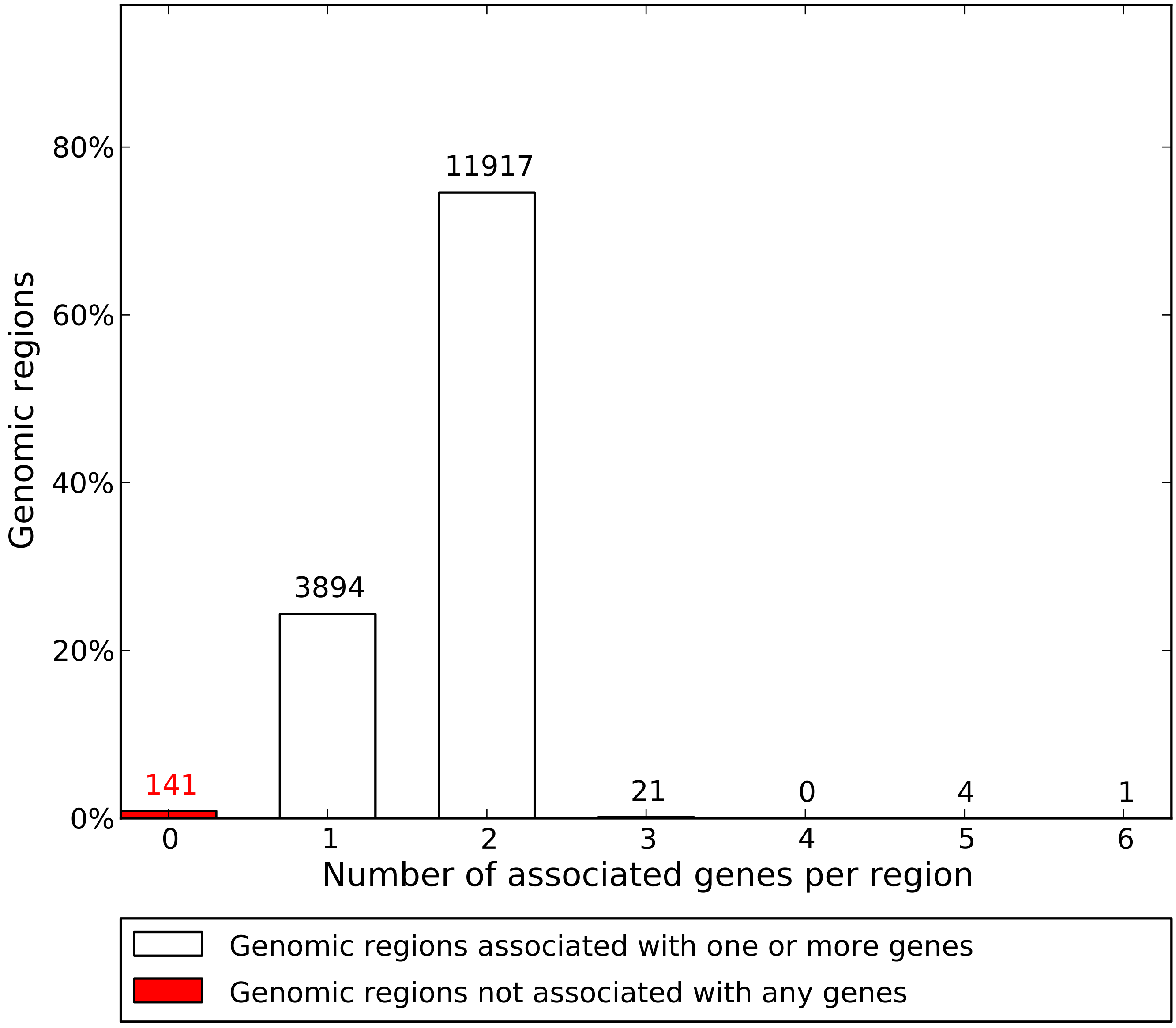
80%
60%
40%
20%
0%
Genomic regions
141
3894
11917
21
0
4
1
0
1
2
3
4
5
6
Number of associated genes per region
Genomic regions associated with one or more genes
Genomic regions not associated with any genes
