# Supplementary figures and images for: Genome-wide DNA methylation and gene expression analyses in monozygotic twins identify potential biomarkers of depression
Source: Transl Psychiatry. 2021 Aug 2;11:416. doi: 10.1038/s41398-021-01536-y (PMC8329295; doi:10.1038/s41398-021-01536-y)

**Module membership vs. gene significance**  
**cor=0.67,  $p < 1e-200$**

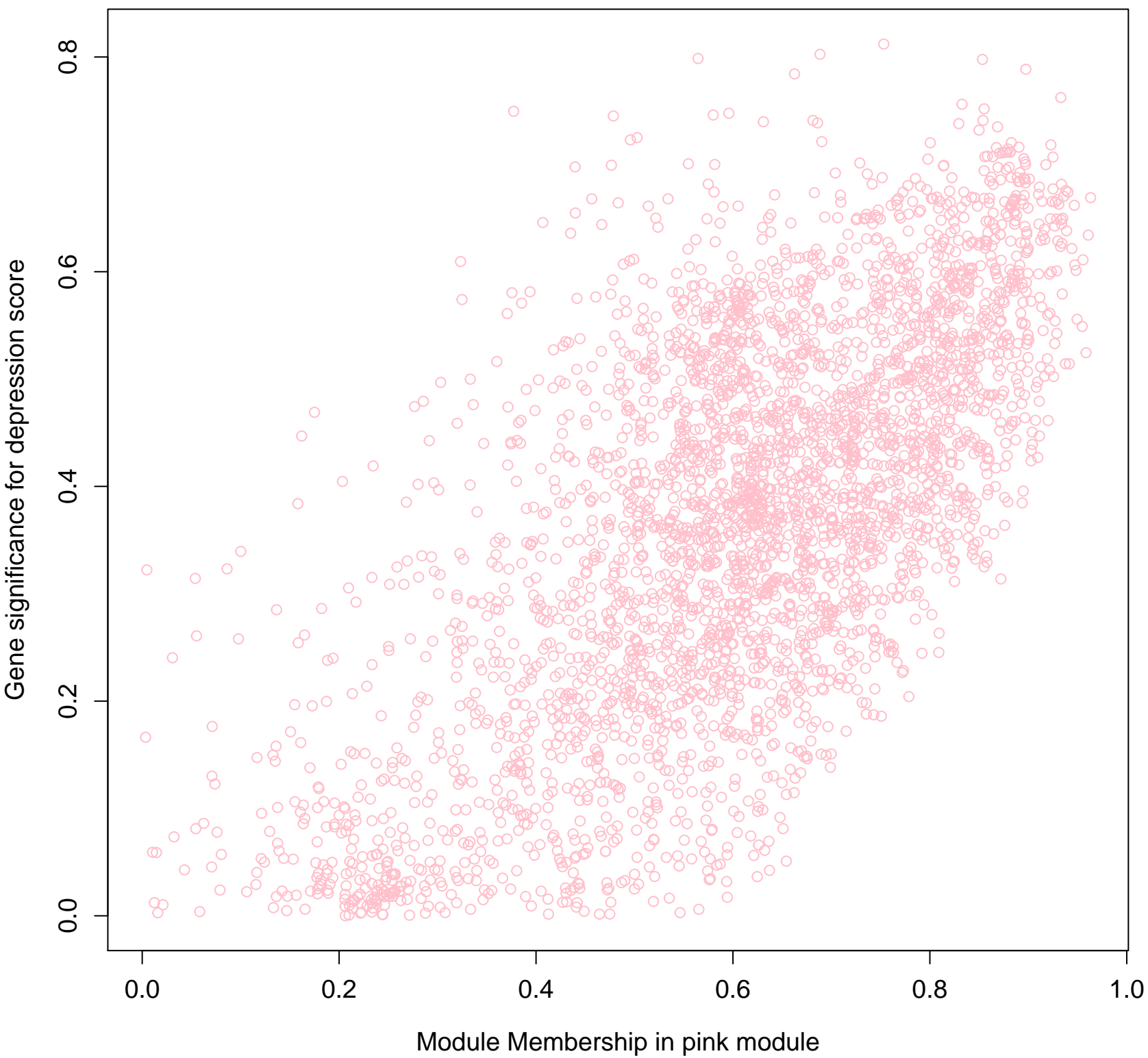

Supplement: Supplementary file 5 — Supplementary figure 2 [file 41398_2021_1536_MOESM5_ESM.pdf]
